# G. lucidum triterpenes restores intestinal flora balance in non-hepatitis B virus-related hepatocellular carcinoma: evidence of 16S rRNA sequencing and network pharmacology analysis

**DOI:** 10.3389/fphar.2023.1197418

**Published:** 2023-09-18

**Authors:** Wei Xiong, Ce Yang, Jing Xia, Wenxiang Wang, Ning Li

**Affiliations:** Chongqing Three Gorges Medical College, Chongqing Key Laboratory of Development and Utilization of Genuine Medicinal Materials in Three Gorges Reservoir Area, Chongqing, China

**Keywords:** non-hepatitis B virus-related hepatocellular carcinoma, ganoderma lucidum triterpenes, Ganosporelactone B, 16S rRNA sequencing, intestinal flora, network pharmacology, CASP3

## Abstract

**Background:** Ganoderma lucidum (G. lucidum) is a popular traditional remedy medicine used in Asia to promote health and longevity, which has also been highlighted for anti-cancer effects. This study investigated the molecular pharmacological mechanism of G. lucidum triterpenes in influencing intestinal flora imbalance in non-hepatitis B virus (HBV)-related hepatocellular carcinoma (HCC) based on 16S rRNA sequencing technology and network pharmacology analysis.

**Methods:** 16S rRNA sequencing data of fecal samples from normal controls and HCC patients were obtained from the SRA database. G. lucidum triterpenes and HCC-related targets were screened by BATMAN-TCM, ETCM, and GeneCards databases. The TCGA-LIHC dataset was downloaded through the TCGA database to analyze the differential expression of key genes. NHBV-related HCC-related transcriptome RNA sequencing dataset was downloaded via the GEO database.

**Results:** Abundance of intestinal flora in the HBV-related HCC and NHBV-related samples was higher than that of control samples. The intestinal flora of NHBV samples was mainly enriched in apoptosis and p53 pathways. Totally, 465 G. lucidum triterpenes-related targets were intersected with 4186 HCC-related targets, yielding 176 intersected targets. Among them, apoptosis and p53 pathway factors were located at the core of the protein-protein interactions network. Ganosporelactone B, the active component of G. lucidum triterpenes, had the lowest binding free energy to CASP3. CASP3 expression were upregulated in HCC tissue samples, and had higher predictive value in NHBV-related HCC patients.

**Conclusion:** Therefore, Ganosporelactone B, the active ingredient of G. lucidum triterpenes, improves the imbalance of intestinal flora and ultimately curtails development of NHBV-related HCC.

## 1 Introduction

Liver cancer is now the sixth most common cancer and the fourth leading cause of cancer death worldwide (McGlynn et al., 2021). In 2020, approximately 905,700 people worldwide will be diagnosed with liver cancer, and 830,200 will die from it, and several new cases of liver cancer are expected to increase by 55.0% per year between 2020 and 2040 (Rumgay et al., 2022). Hepatocellular carcinoma (HCC) (75%–85% of cases) is the main type of primary liver cancer and one of the major public health problems worldwide (Forner et al., 2018). The main risk factors for HCC are hepatitis B virus (HBV), hepatitis C virus (HCV), hepatitis B virus (HCV), cirrhosis, smoking, excessive alcohol consumption, aflatoxin exposure, obesity, and type 2 diabetes mellitus. The key determinants in most high-risk areas (e.g., China and Africa) are chronic HBV infection and aflatoxin exposure. HBV infection is the most strongly associated, accounting for 75%–80% of virus-related HCC (2021). However, with hepatitis B vaccination, their importance is likely to decline in the coming years (Xie, 2017). Unfortunately, the prevalence of other risk factors inducing HCC, including metabolic syndrome and obesity, is increasing and may become a major cause of HCC development globally (Zhang C H et al., 2022). Thus, it is also valuable to investigate the specific mechanisms underlying the occurrence and development of non-HBV (non-HBV, NHBV)-related HCC (NHBV-related HCC), which may provide new ideas to improve the treatment outcome and prognosis of HCC.

Currently, surgery remains the treatment of choice for HCC. However, as most patients with HCC already have cirrhosis or have reached an intermediate to advanced stage at the time of presentation, only 10%–20% of patients meet the criteria for complete surgical resection (Torimura and Iwamoto, 2022). In addition, the high recurrence rate after surgery remains the most serious challenge after surgical resection, with a 5-year survival rate of only 30%–40% (Salem et al., 2022). Several treatment options are available for HCC depending on liver function, tumor size, number, vascular invasion, or degree of extrahepatic spread, but all have very limited therapeutic efficacy (Anwanwan et al., 2020). With the development of science and technology, traditional Chinese medicine’s pharmacological effects and mechanisms of action have been further revealed and demonstrated (Xiang et al., 2019). Natural compounds, active ingredients, single herbs, and compound formulas can preferentially kill cancer cells and inhibit their expansion without obvious toxicity (Yang et al., 2021). According to statistics, 80% of primary HCC patients in China have been treated with Chinese herbal medicine to varying degrees (Liu C et al., 2019; Liu J S et al., 2019). Traditional Chinese medicine or natural compounds have good potential for application in tumor treatment, such as HCC.

Ganoderma lucidum (G. lucidum, Lingzhi or Reishi) is a medicinal mushroom historically used in Asian countries to treat a wide variety of diseases and prolong life (Gurovic et al., 2018). It has anti-cancer effects according to previous study (Kladar et al., 2016). G. lucidum triterpenoids (GLTs) are a group of triterpenoids found in G. lucidum, scientifically known as G. lucidum total triterpenes, which are highly oxidized lanolin derivatives and one of the main chemical and pharmacological components of G. lucidum (Cor et al., 2018). Available data indicate that G. lucidum triterpenes have many anticancer properties, including anti-proliferative, anti-metastatic, and anti-angiogenic activities (Wu et al., 2013). G. lucidum triterpenes contain erythranilic acid, ganoderic acid, ganoderma lactone, and ganoderol. Due to the continuous improvement in extraction techniques, the unit content of G. lucidum triterpenes has been increased to achieve significant medicinal effects (Koo et al., 2021). It has been shown that G. lucidum triterpenes are “good at attacking” and can directly damage tumor cell DNA (Gurovic et al., 2018). G. lucidum triterpene extracts can cause cell cycle arrest and inhibit the growth of liver cancer cells (Lin et al., 2003). G. lucidum triterpenes may play an important role in the treatment of HCC. This study will further investigate the key active components, key targets, and key pathways of G. lucidum triterpenes in the treatment of HCC.

There is a functional link between the liver and the intestine, with the liver being the first barrier organ against bacteria of the intestinal origin or their metabolites (Szabo, 2015). Dysregulation of intestinal ecology has been found in mouse models of cirrhosis, HCC patients, and liver cancer (Zeng et al., 2020). Much evidence has confirmed that alterations in the intestinal microbiota or barrier function are key factors in promoting the development of chronic liver disease to HCC (Behary et al., 2021; Schneider et al., 2022). It is worth mentioning that Chinese medicine can improve the intestinal microbiota and the intestinal mucosal barrier function, which suggests that Chinese medicine can have therapeutic effects on tumor patients by regulating the balance of intestinal flora (Chen Y Z et al., 2021; Zhao et al., 2021). At the same time, G. lucidum triterpenes had a regulatory effect on intestinal flora in rats on a high-fat diet (Tong et al., 2023). We, therefore, hypothesize that G. lucidum triterpenes may treat HCC by improving the imbalance of intestinal flora.

This study aimed to investigate the key mechanism of G. lucidum triterpenes in treating HCC by improving the imbalance of intestinal flora based on 16S rRNA sequencing, network pharmacology and network pharmacology, and bioinformatics technologies. This study is expected to provide new evidence for the prognosis of HCC patients treated with G. lucidum triterpenes and new ideas for the future precision treatment of NHBV-related HCC patients.

## 2 Materials and methods

### 2.1 16S rRNA sequencing data acquisition

Phenotype information for all samples from the HCC-related project was retrieved through the EMBL-EBI database (https://www.ebi.ac.uk/ena/browser/search) (BioProject number: PRJNA428932). 16S rRNA sequencing data for the project samples were further downloaded via the NCBI Sequence Read Archive (SRA) database (https://www.ncbi.nlm.nih.gov/sra/), including 33 healthy control fecal samples, 22 fecal samples from non-HBV-related HCC patients and 35 fecal samples from HBV-related HCC patients. All data used in the study were obtained from publicly available databases and therefore did not require ethics committee approval.

### 2.2 Analysis of intestinal flora diversity and species composition

Samples were assessed by applying multiQC and kneaddata (https://github.com/biobakery/biobakery/wiki/kneaddata). multiQC was used for sequence quality control, and kneaddate was used to remove host and contaminating sequences. Relative abundance of microbial taxa was obtained by mapping microbial species trees and annotating differences by GraPhlAn (https://github.com/biobakery/graphlan.git). To assess the diversity of species complexity in the samples, Alpha diversity analysis was performed by Richness index and Chao1 index, and Beta diversity was analyzed by Constrained Principal Coordinates Analysis (CPCoA) (Jiang et al., 2021). Wilcoxon rank sum and Welch t-test were used to comparing bacterial abundance and diversity. Differences in abundance between groups were calculated using the R package edgeR, and volcano and Manhattan plots were plotted. All difference abundance histograms were plotted by LDA Effect Size (LEfSe, http://huttenhower.sph.harvard.edu/lefse/) analysis, with a linear divergence analysis (LDA) score threshold set at 2.0. LDA scores indicate the degree of effect of significantly different species between groups, with higher scores indicating higher scores indicate greater differences in characteristics between the two groups.

### 2.3 Analysis of the functional composition of the intestinal flora

QIIME data were transformed using R package Compositions followed by Phylogenetic Investigation of Communities by Reconstruction of Unobserved States States (PICRUSt) was used to predict metagenome pathways for each primer set using the Kyoto Encyclopedia of Genes and Genomes (KEGG) (Kanehisa et al., 2016; Douglas et al., 2020). The unstratified results were statistically analyzed and visualized using STAMP (v2.1.3) software. Welch t-tests were used to compare differences in functional composition.

### 2.4 Access to G. lucidum triterpenes and HCC-related targets

The active ingredients of G. lucidum triterpenes were obtained through the TCMSP database (https://tcmsp-e.com/tcmsp.php). The targets corresponding to the active ingredients of G. lucidum triterpenes were searched individually through the BATMAN-TCM database (http://bionet.ncpsb.org.cn/batman-tcm/). The targets corresponding to the active ingredients of G. lucidum triterpenes were also retrieved individually through the ETCM database (http://www.tcmip.cn/ETCM/index.php/Home/Index/). The screening results of BATMAN-TCM and ETCM databases were merged, and duplicate genes were removed, which were the G. lucidum triterpenes-related targets.

The GeneCards database (https://www.genecards.org/) was searched for “hepatocellular carcinoma,” and the corresponding targets were downloaded. Differentially expressed genes in the HCC dataset (TCGA-LIHC) were screened by the GEPIA database (http://gepia2.cancer-pku.cn/#index). The GeneCards and GEPIA database screening results, were merged, and duplicate genes were removed, resulting in HCC-related targets.

### 2.5 Cross-target acquisition and KEGG enrichment analysis

Using the jvenn tool (http://jvenn.toulouse.inra.fr/app/index.html), the G. lucidum triterpenes-related targets were intersected with the HCC-related targets to obtain the intersected targets. KEGG enrichment analysis of the intersecting targets was performed using the R package clusterProfiler, and bar graphs were plotted. In addition, we obtained maps of apoptosis pathway regulatory mechanisms (https://www.kegg.jp/pathway/hsa04210) and p53 pathway regulatory mechanisms through the KEGG database (https://www.kegg.jp/kegg/).

### 2.6 Protein interaction network construction

The intersection targets of G. lucidum triterpenes and HCC were imported into the STRING database (https://string-db.org) with the species restriction “*Homo sapiens*” to obtain the protein interaction network. The regulatory network was further imported into Cytoscape (v3.8.2) software to visualize the network relationship map and to tag Apoptosis and p53 pathway-related factors.

In addition, we imported the component-target correspondence and the target-pathway correspondence into Cytoscape (v3.8.2) software to further construct the “G. lucidum triterpenes component-target-pathway” network.

### 2.7 G. lucidum triterpenes small molecule ligand file preparation

The 2D structures of the small molecule ligands of each of the 10 G. lucidum triterpenes components were downloaded via the PubChem database (https://pubchem.ncbi.nlm.nih.gov/), followed by conversion of the 2D structures into 3D structures using ChemBio3D (v14.0) software, while the 3D structures were optimized using the MM2 algorithm with minimum free energy. The mol2 format file was exported. The small molecule ligands were further saved as “pdbqt” files using AutoDockTools (v1.5.7) (Li et al., 2022).

### 2.8 Protein receptor file preparation

Crystal structures of 13 Apoptosis and p53 pathway-related target proteins screened from the Protein Data Bank database (https://www.rcsb.org) were downloaded, specifically: NFKBIA (PDB ID: 1ikn), NFKB1 (PDB ID: 6ttu), BAX (PDB ID: 5w61), CASP3 (PDB ID: 1rhq), BAK1 (PDB ID: 2jcn), BCL2 (PDB ID: 2w3l), TNF (PDB ID: 1a8m), AKT1 (PDB ID: 1h10), CCND1 (PDB ID: 2w9z), CCNE1 (PDB ID: 1w98), IGF1 (PDB ID: 1b9g), PTEN (PDB ID: 7juk), CDKN1A (PDB ID: 2zvv). These 13 target protein receptors were processed using PyMOL (v2.5.4) software for dehydration and organic matter removal to derive protein molecules. The receptor protein molecules were hydrogenated, and the charge was calculated using AutoDockTools (v1.5.7) and saved as “pdbqt” files to determine the active pocket of the receptor protein (Seeliger and de Groot, 2010).

### 2.9 Molecular simulation docking

Molecular docking of the above-prepared G. lucidum triterpenes small molecule ligands and protein receptors was performed by Vina (v1.5.7) software to calculate the Binding Affinity and derive a molecular docking schematic (Seeliger and de Groot, 2010).

### 2.10 TCGA data download and clinical data analysis

Download the TCGA-LIHC dataset through The Cancer Genome Atlas (TCGA) database (https://portal.gdc.cancer.gov/) to download data related to the TCGA-LIHC dataset, which contains 50 normal liver tissue samples and 3 RNA sequencing data from 74 cancer tissue samples from HCC patients (HTSeq-FPKM) and survival information from 371 HCC patients. Patients were divided into high and low-expression groups based on median values of key gene expression. Kaplan-Meier survival analysis was performed to compare the difference in overall survival (OS) between the two groups, with *p* < 0.05 selected as the cut-off value.

### 2.11 GEO data download and clinical data analysis

The HBV-related HCC-related dataset GSE10143 was downloaded via the Gene Expression Omnibus (GEO) database (https://www.ncbi.nlm.nih.gov/geo/). It contained 307 normal liver tissue samples, 61 samples from NHBV patients, and 19 HBV patient liver tissue samples. The difference in CASP3 expression between the three groups was analyzed using an unpaired t-test, with *p* < 0.05 indicating a statistically significant difference. Patients were divided into high and low-expression groups according to the median value of CASP3 expression. Kaplan-Meier survival analysis was performed to compare the difference in overall survival (OS) between the two groups, with *p* < 0.05 selected as the cut-off value. ROC analysis was performed using the R package, and ROC curves were plotted to assess the predictive value of CASP3. ROC curves and the area under the ROC curve (AUC values) were used to assess the sensitivity and specificity of the survival analysis model.

## 3 Results

### 3.1 Significant differences in intestinal flora diversity between normal controls and HCC patients and more significant differences between NHBV-related HCC than HBV-related HCC

We obtained 16S rRNA sequencing data from fecal samples of normal controls and HCC patients through the SRA database (BioProject number: PRJNA428932), where HCC patients were divided into HBV-related HCC group (HBV group) and NHBV-related HCC group (NHBV group).

**FIGURE 1:**
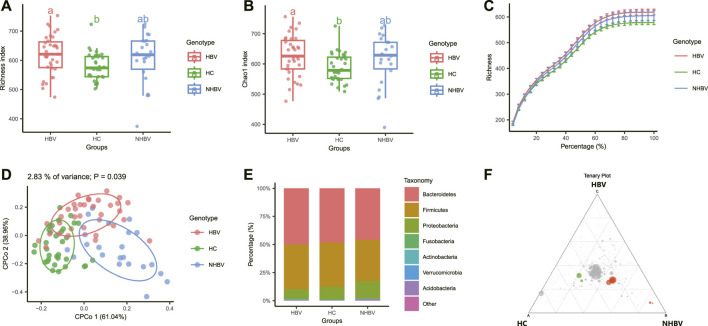
Comparison of intestinal flora species diversity between normal control and HCC patients. Note: **(A–B)** Analysis of alpha diversity of intestinal flora in normal control and HCC patients, Figure A shows Richness index, Figure B shows Chao1 index; **(C)** alpha sparsity curve of intestinal flora in normal control and HCC patients; **(D)** analysis of beta diversity of intestinal flora in normal control and HCC patients; **(E)** stacked histogram with the group as horizontal axis at the gate **(E)** stacked histogram of relative abundance at the gate (Phylum) level with the group as the horizontal axis; **(F)** Tenary plot comparing the three groups of differential flora, green for the HC group, red for the NHBV group and blue for the HBV group (not shown in the plot as the difference was not significant); HC group, *n* = 33; NHBV group, *n* = 22; HBV group, *n* = 35.

Alpha diversity is a method to calculate the species composition within a sample, including both quantitative and abundance information. We found the alpha diversity indices. Richness index and Chao1 index significantly differed between HCC patients (NHBV and HBV groups) and normal control (HC group) fecal samples. In addition, there were also significant differences between the NHBV and HBV groups (Figures 1A, B). The results of the Rarefraction Curve further showed that the abundance of intestinal flora was significantly higher in HCC patients (NHBV and HBV groups) than in normal controls (HC group) (Figure 1C).

Beta diversity is the study of differences in species composition between communities. We calculated the Beta diversity distance matrix using three common similarity/distance indices (bray_curtis, jaccard, and manhatten) and conducted Constrained Principal Coordinates Analysis (CPCoA) based on bray_curtis distances. Analysis (CPCoA) based on the bray_curtis distance found a significant separation between the three sample groups (Figure 1D).

Further species composition analysis was performed, and the R package edgeR was used to calculate differences in intestinal flora abundance between the three groups, and volcano, heat, and Manhattan plots were plotted, showing that at the phylum level, there were significant differences in operational taxonomic units (OTUs) between the NHBV and HBV groups compared to the HC group, with NHBV There were 119 significantly Depleted OTUs and 75 significantly Enriched OTUs in the NHBV group and 87 significantly Depleted OTUs and 27 significantly Enriched OTUs in the HBV group (Supplementary Figure S1).

In addition, our preliminary analysis of species composition at the phylum (Phylum) level showed that the species composition of the gut flora of HCC patients (NHBV group and HBV group) and normal controls (HC group) differed significantly at the phylum (Phylum) level, with Bacteroidetes being more abundant in the HBV group and Proteobacteria being more abundant in the NHBV group were more abundant, which is consistent with evidence from the literature (Chen Y et al., 2021; Zhang C H et al., 2022) (Figure 1E). A Tenary plot was also plotted, and as shown in Figure 1F, there was a clear separation of the three groups, with more differential colonies in the HC and NHBV groups and less in the HBV group.

### 3.2 Significant differences in the species composition of the intestinal flora between normal controls and HCC patients

**FIGURE 2:**
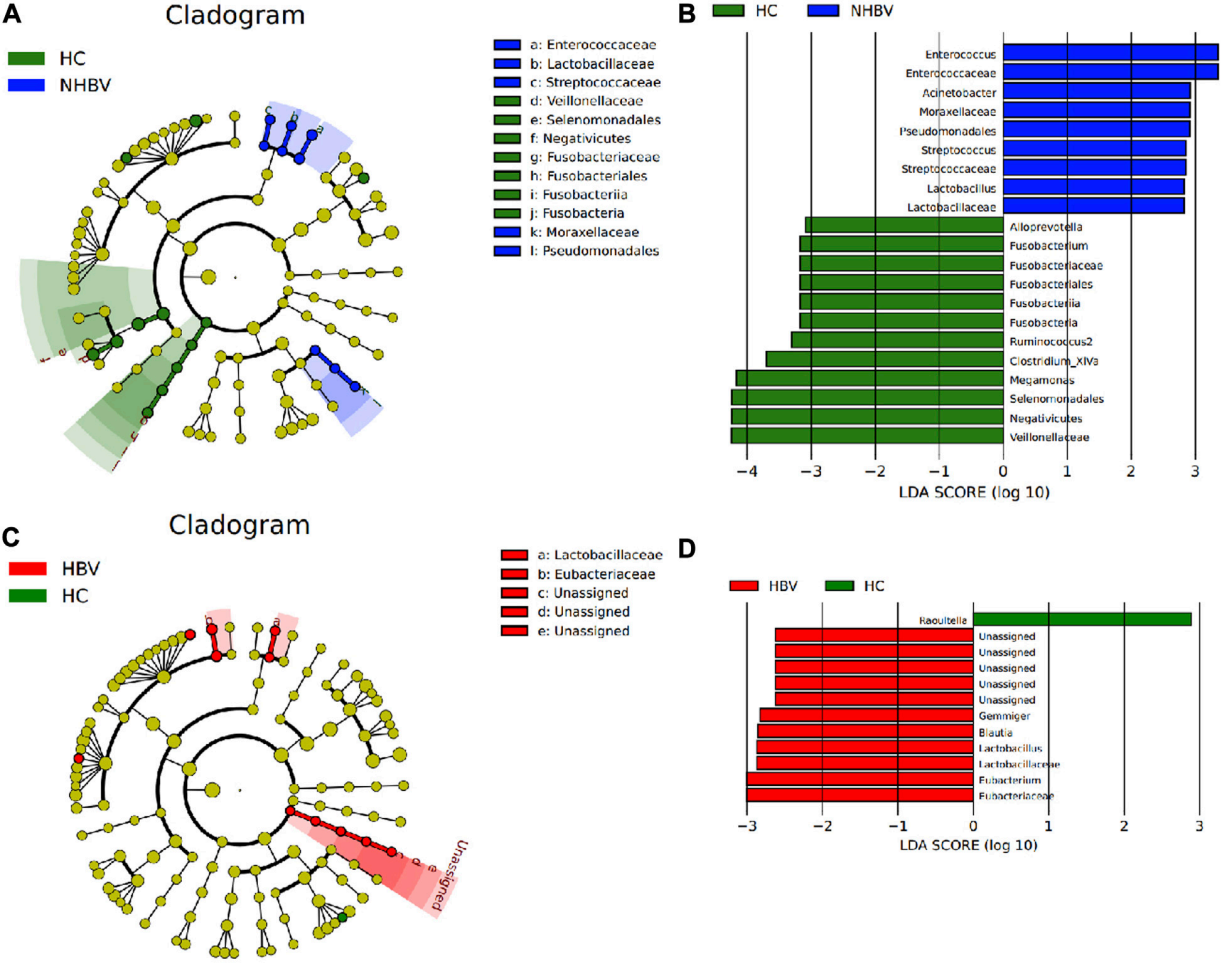
LEfSe analysis of differences in species composition of the intestinal flora of normal controls and HCC patients at the genus level. Note: **(A)** Taxonomic branching plots of species abundance of intestinal flora in the HC and NHBV groups; **(B)** histograms of the distribution of LDA values for species abundance of intestinal flora in the HC and NHBV groups; **(C)** taxonomic branching plots of species abundance of intestinal flora in the HC and HBV groups; **(D)** histograms of the distribution of LDA values for species abundance of intestinal flora in the HC and HBV groups; the circles radiating from inside to outside in Figs. A and C circles in Figures A and C represent taxonomic levels from species to genus, and the size of the diameter represents the relative abundance; yellow nodes indicate species with no significant differences, green nodes indicate microbial taxa with higher abundance in the HC group, blue nodes indicate microbial taxa with higher abundance in the NHBV group, and red nodes indicate microbial taxa with higher abundance in the HBV group; green bars in Figures B and D indicate microbial taxa with higher abundance in the HC group The green bars in Fig. B and D indicate microbial taxa that were more abundant in the HC group, the blue bars indicate microbial taxa that were more abundant in the NHBV group, and the red bars indicate microbial taxa that were more abundant in the HBV group; HC group, *n* = 33; NHBV group, *n* = 22; HBV group, *n* = 35.

To investigate the differences in species composition of the intestinal flora between the three groups, we narrowed it down to the genus (Genus) level and performed LEfSe analysis. *Acinetobacter*, Moraxellaceae, Pseudomonadales, *Streptococcus*, Streptococcaceae, *Lactobacillus*, and Lactobacillaceae were significantly more abundant in fecal samples from patients in the NHBV group than in the HC group, while the relative abundance of Alloprevotella, *Fusobacterium*, Fusobacteriaceae, Fusobacteriales, Fusobacteria, Fusobacteria, Ruminococcus2, *Clostridium*_ XIVa, Megamonas, Selenomonadales, Negativicutes, Veillonellaceae were significantly more abundant in relative terms than the NHBV group (Figures 2A, B). In addition, the relative abundance of Gemmiger, Blautia, *Lactobacillus*, Lactobacillaceae, Eubacterium, and Eubacteriaceae in the fecal samples of patients in the HBV group was significantly higher than that in the HC group, while the relative abundance of Raoultella in the fecal samples of patients in the HC group was significant. The relative abundance of Raoultella in fecal samples from patients in the HC group was significantly higher than that in the HBV group (Figures 2C, D).

It has been shown that Proteobacteria increased and Ruminococcus decreased in the intestinal flora of HCC patients in the presence of a vascular invasion group compared to the absence of a vascular invasion group (Zhang N et al., 2022), which is also consistent with the results of the analysis in the present study. Therefore, we conclude that there are significant differences in the composition of the intestinal flora between normal controls and HCC patients at the genus (Genus) level and that these differentially enriched microbiota are sufficient to distinguish between the microbiota in fecal samples from normal controls and HCC patients.

### 3.3 Apoptosis and p53 pathways may be key pathways through which intestinal flora influence the development and progression of NHBV-Related HCC

**FIGURE 3:**
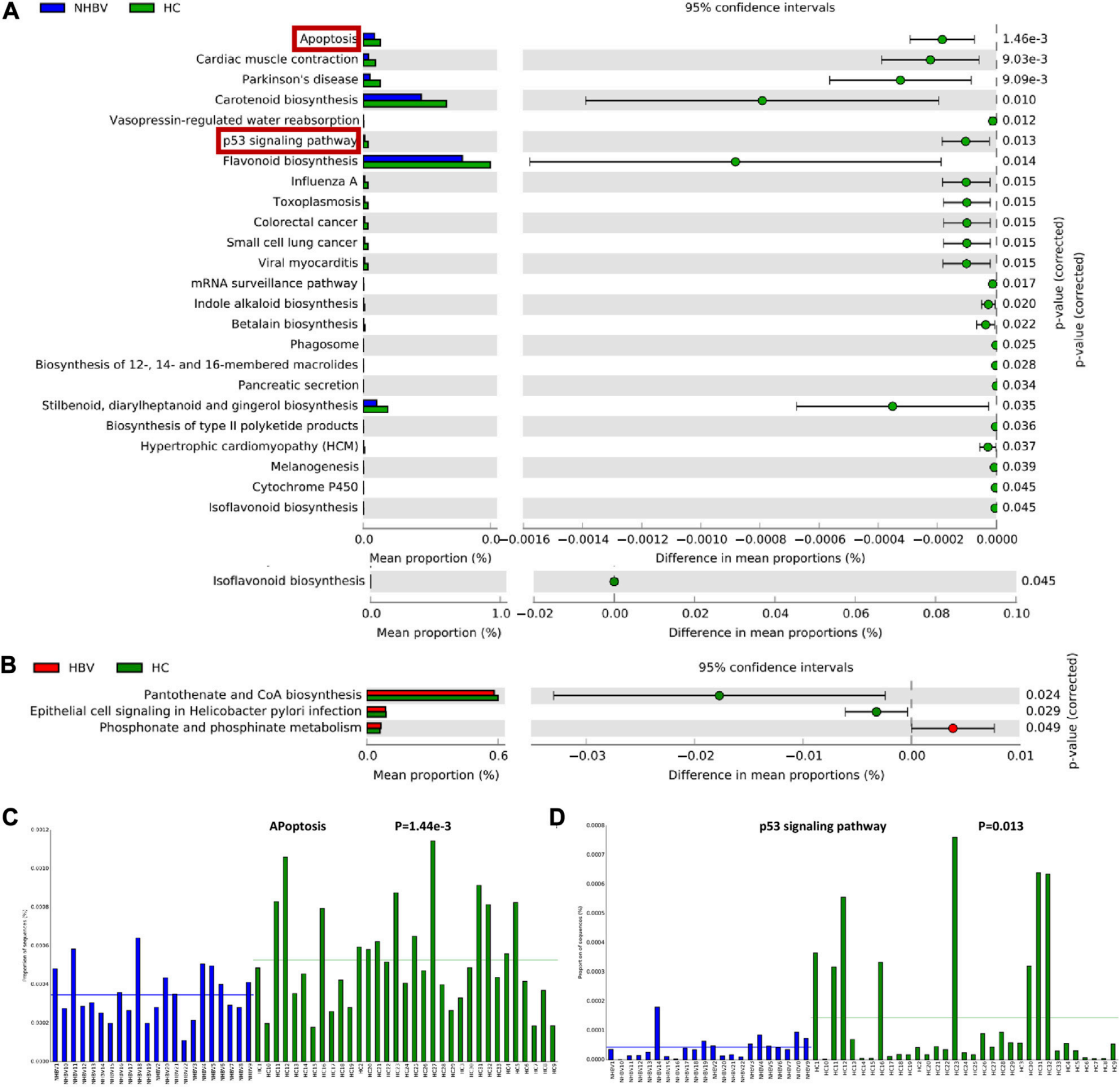
Functional composition of the intestinal flora of normal controls and HCC patients. Note: **(A)** Functional enrichment analysis of gut flora in HC and NHBV groups; **(B)** Functional enrichment analysis of gut flora in HC and HBV groups; **(C)** Enrichment of differential gut flora in apoptosis pathway in HC and NHBV groups; **(D)** Enrichment of differential gut flora in p53 pathway in HC and NHBV groups; green bars in Figure A and B indicate functional pathways significantly enriched in the HC group, blue bars indicate functional pathways significantly enriched in the NHBV group and red bars indicate functional pathways significantly enriched in the HBV group; HC group, *n* = 33; NHBV group, *n* = 22; HBV group, *n* = 35.

To investigate the differences in the functional composition of the intestinal flora between HCC and normal control patients, we performed a KEGG enrichment analysis of the intestinal flora based on Genus (genus) abundance data by PICRUSt2 software. Further, we visualized the functional composition of the intestinal flora by STAMP software. Our analysis showed that the differential intestinal flora between the NHBV and HC groups were mainly enriched in Apoptosis, Cardiac muscle contraction, Parkinson’s disease, Carotenoid biosynthesis, Vasopressin-regulated water reabsorption, p53 pathway, Flavonoid biosynthesis, Influenza A, Toxoplasmosis, Colorectal cancer, Small cell lung cancer and other functional pathways (Figure 3A). The difference in intestinal flora between HBV and HC groups was mainly enriched in Pantothenate and CoA biosynthesis, Epithelial cell signaling in *Helicobacter pylori* infection, and Phosphonate and phosphinate metabolism functional pathways (Figure 3A). Phosphinate metabolism functional pathways (Figure 3B).

It has been well documented that Apoptosis and p53 pathways play an important role in tumors such as HCC (Meng et al., 2014; Liu X et al., 2019; Lan et al., 2021). As shown in Figures 3C, D, apoptosis and p53 pathways were significantly less enriched in the NHBV group compared to the HC group. Therefore, we hypothesized that: Apoptosis and p53 pathways may be key pathways through which intestinal flora affect the occurrence and development of NHBV-related HCC.

### 3.4 G. lucidum triterpenes improve intestinal flora imbalance in NHBV-Related HCC patients by activating apoptosis and p53 pathways

Previous evidence shows that G. lucidum can improve intestinal flora dysbiosis and reduce AOM/DSS-induced colitis and tumorigenesis by inhibiting TLR4/MyD88/NF-κB pathway (Guo et al., 2021). As the main active component of G. lucidum to exert anti-tumour effects, G. lucidum triterpenes have good potential for application in tumor therapy (Wu et al., 2013). It has been shown that G. lucidum triterpenes have a modulating effect on intestinal flora in rats on a high-fat diet (Tong et al., 2023). Thus, we hypothesize that G. lucidum triterpenes may treat HCC by improving the imbalance of intestinal flora.

Firstly, we obtained 28 active ingredients of G. lucidum triterpenes through the TCMSP database. Further, we searched the BATMAN-TCM database for each of the 28 active ingredients and obtained 415 targets with a threshold of Score >5.0. The ETCM database was also searched for targets for each of the 28 active ingredients, using a threshold of Score >0.8 (the default threshold), and 108 targets were obtained. The screening results of BATMAN-TCM and ETCM databases were merged, and duplicate genes were removed to obtain 465 G. lucidum triterpenes-related targets.

**FIGURE 4:**
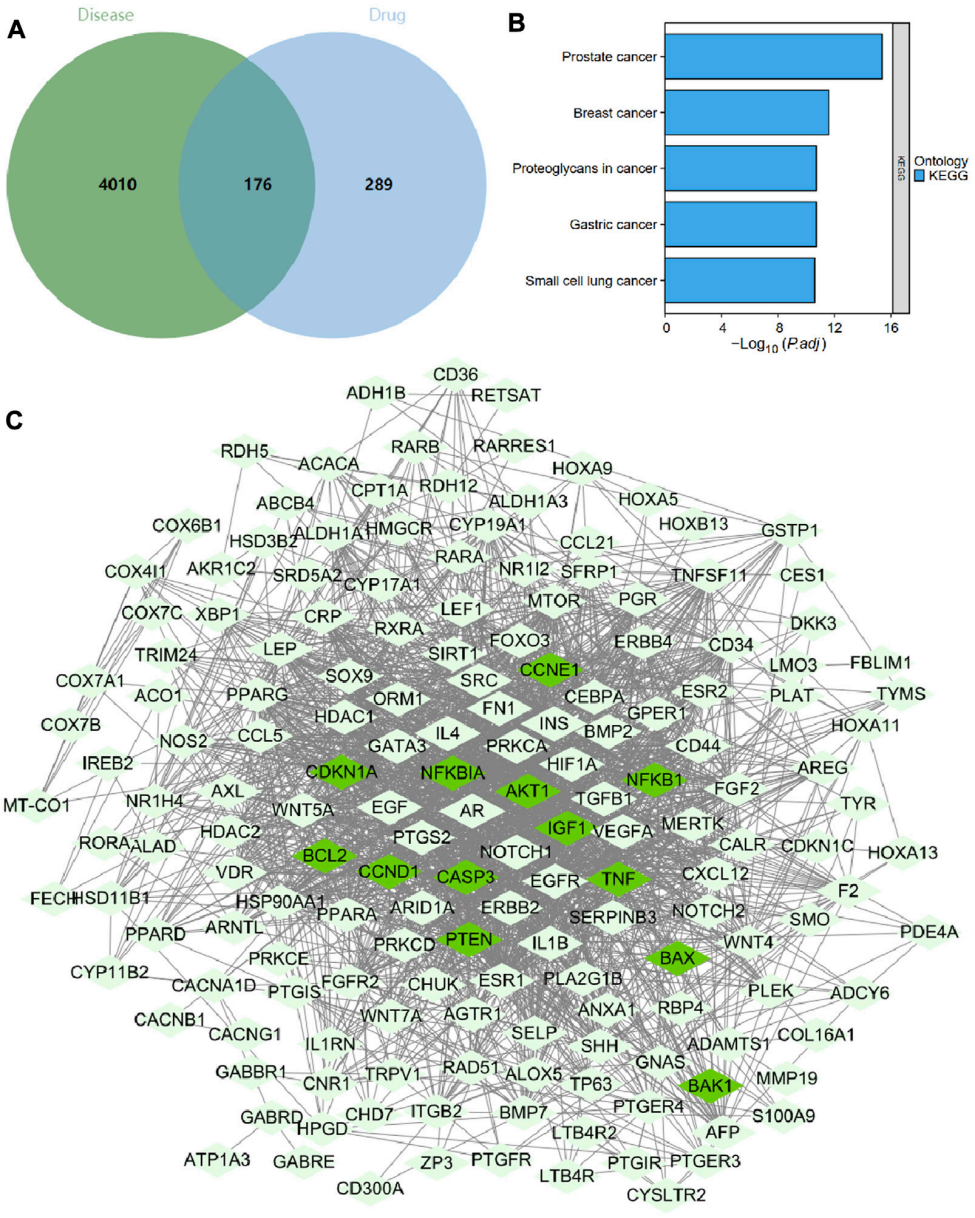
Screening, network construction, and functional enrichment analysis of key targets of G. lucidum triterpenes. Note: **(A)** Venn diagram of HCC patient-related targets intersected with G. lucidum triterpenes-related targets; **(B)** KEGG enrichment analysis results of 176 intersected targets; **(C)** network diagram of 176 intersected target-encoded proteins interacting with each other, with 13 Apoptosis and p53 pathway factors in dark green nodes.

Meanwhile, the GeneCards database was searched for “hepatocellular carcinoma”, and 1201 genes were screened using a threshold of Relevance score >10. The GEPIA database was searched for differentially expressed genes in the HCC dataset (TCGA-LIHC) with a threshold of |Log_2_ FC| > 1 and *p* < 0.05, and 3202 genes were screened. The GeneCards and GEPIA database screening results, were taken and merged, and duplicate genes were removed to obtain 4186 HCC-related targets. We then intersected the 465 G. lucidum triterpenes-related targets with the 4186 HCC-related targets to obtain 176 intersected targets (Figure 4A). The KEGG enrichment analysis revealed that these 176 intersecting targets were mainly enriched in the tumor pathway, suggesting that G. lucidum triterpenes have good potential for application in tumor therapy (Figure 4B).

We imported 176 intersecting targets into the String database, limiting the species to humans to obtain protein interaction relationships. We found that Apoptosis and p53 pathway factors (dark green nodes) were located at the core of the protein interaction network (Figure 4C). The specific regulatory mechanisms of these 13 targets (NFKBIA, NFKB1, BAX, CASP3, BAK1, BCL2, TNF, AKT1, CCND1, CCNE1, IGF1, PTEN, CDKN1A) in the apoptosis and p53 pathways are shown in Supplementary Figures S2, S3 shows. Therefore, we hypothesize that G. lucidum triterpenes may improve the imbalance of intestinal flora by activating apoptosis and p53 pathways and ultimately prevent the occurrence and development of NHBV-related HCC.

### 3.5 G. lucidum triterpene small molecule ligands act by targeting and binding to six key target protein receptors

**FIGURE 5:**
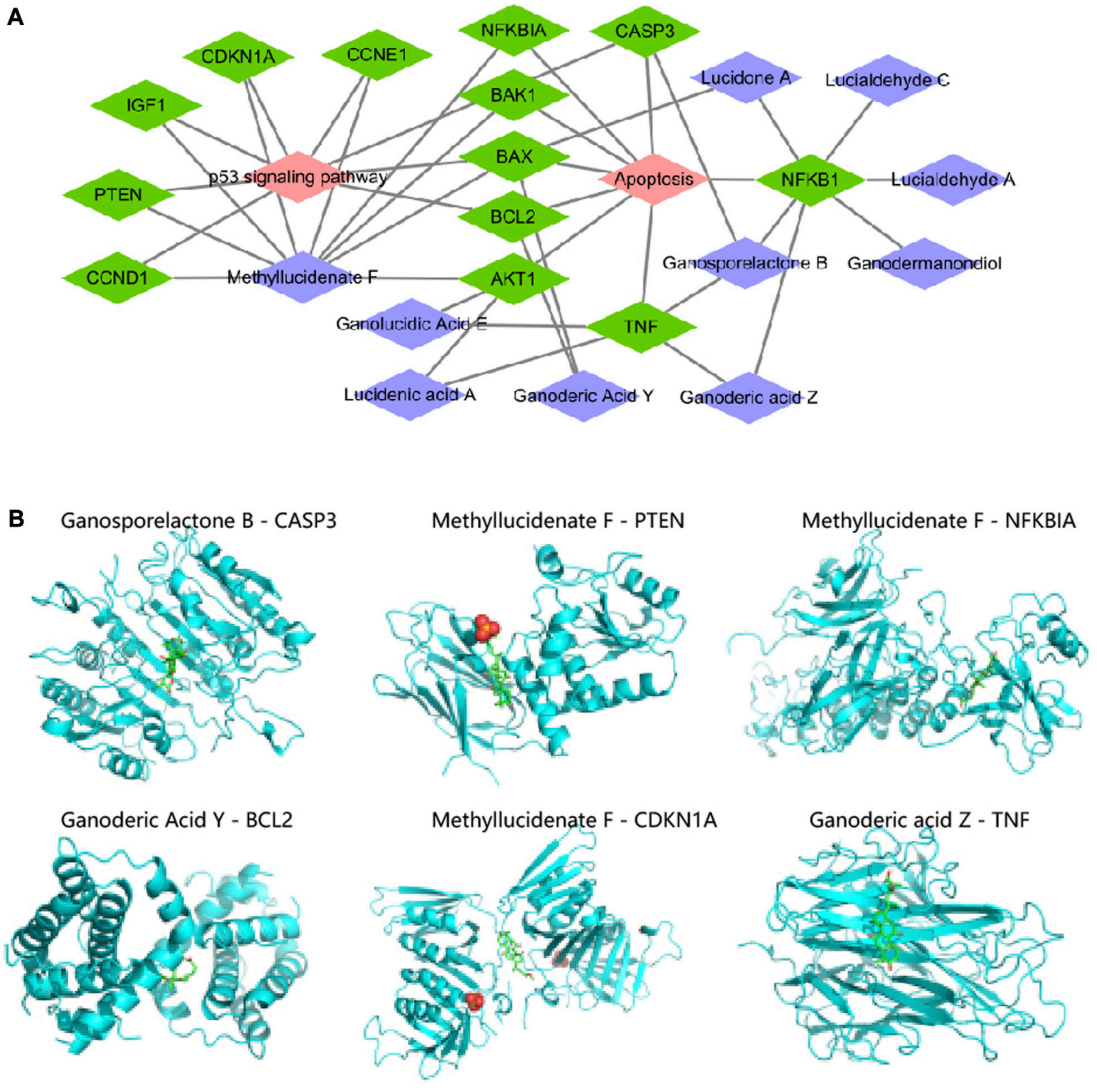
“G. lucidum triterpenes component-target-pathway” network construction and molecular docking. Note: **(A)** “G. lucidum triterpenes component-target-pathway” network constructed by Cytoscape software, in which purple nodes indicate G. lucidum triterpenes components, green nodes indicate targets, and red nodes indicate pathways; **(B)** Molecular docking of each small molecule ligand and protein receptor, in which blue part is protein receptor and green **(B)** Molecular docking of each small molecule ligand to the protein receptor.

Based on the “component-target” relationships in the BATMAN-TCM and ETCM databases, we obtained 13 Apoptosis and p53 pathway-related targets (NFKBIA, NFKB1, BAX, CASP3, BAK1, BCL2, TNF, AKT1, CCND1, CCNE1, IGF1, PTEN, CDKN1A CCND1, CCNE1, IGF1, PTEN, CDKN1A) corresponding to the 10 G. lucidum triterpenes components (Ganodermanondiol, Lucialdehyde C, Ganoderic acid Z, Lucialdehyde A, Methyllucidenate F, Lucidone A, Ganosporelactone B, Ganoderic Acid Y, Lucidenic acid A, Ganolucidic Acid E), and further constructed the “G. lucidum triterpenes Component-Target-Pathway” network relationship (Figure 5A). The specific information on the 10 G. lucidum triterpenes is shown in Supplementary Table S1.

The activity of many drugs and other biomolecules is expressed through interactions with receptor macromolecules. Evaluating the Binding Affinity between receptors and ligands is a central issue in structure-based computer-aided drug molecular design (Pantsar and Poso, 2018). We then prepared 10 small molecule ligand files for G. lucidum triterpenes components and 13 protein receptor files for Apoptosis and p53 pathway-related targets, all saved as “pdbqt” files, to determine the active pockets of the receptor proteins, and then performed molecular docking using Vina software. The molecular ligands were considered able to bind and interact with the protein receptors when the free energy of binding was <0 kJ/mol. The lower the free energy of binding, the more stable the molecular conformation (Pilipovic et al., 2021).

As shown in Supplementary Table S2, we have ranked the binding free energies in descending order, Ganosporelactone B with CASP3 (−9.3 kal/mol), Methyllucidenate F with PTEN (−9.0 kal/mol), Methyllucidenate F with NFKBIA (−8.9 kal/mol), Ganoderic Acid Y with BCL2 (−8.6 kal/mol), Methyllucidenate F with CDKN1A with NFKBIA (−8.9 kal/mol), Ganoderic Acid Y with BCL2 (−8.6 kal/mol), Methyllucidenate F with CDKN1A (−8.1 kal/mol), Ganoderic acid Z with TNF (−8.0 kal/mol) all had binding free energies of ≤ −8.0 kcal/mol (Figure 5B). The docking of the other 19 small molecule ligands with protein receptors is shown in Supplementary Figure S4. Thus, we suggest that CASP3, PTEN, NFKBIA, BCL2, CDKN1A, and TNF may be the key targets of Ganoderic acid triterpenes in influencing the development and progression of NHBV-related HCC.

### 3.6 CASP3 may be a key gene mediating the imbalance of intestinal flora for the development and progression of NHBV-related HCC

**FIGURE 6:**
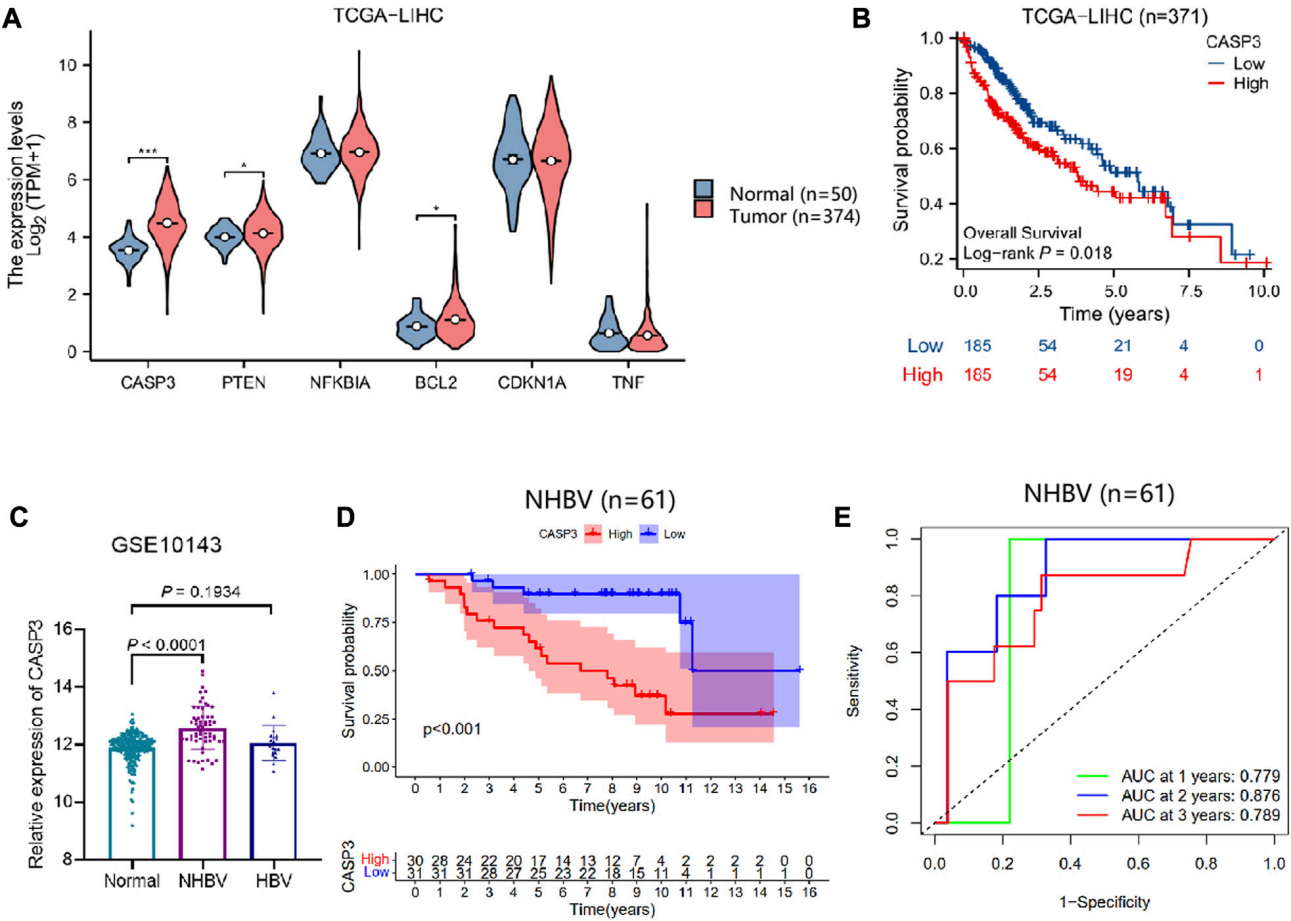
Correlation between CASP3 and survival of NHBV-related HCC patients. Note: **(A)** Analysis of CASP3, PTEN, NFKBIA, BCL2, CDKN1A, TNF expression in normal control and HCC patients based on TCGA-LIHC dataset, * indicates *p* < 0.05, *** indicates *p* < 0.001 (Normal group, *n* = 50; Tumor group, *n* = 374) **(B)** Analysis of the correlation between CASP3 and overall survival of HCC patients by plotting survival curves (*n* = 371); **(C)** Analysis of the differential expression of CASP3 in the Normal, NHBV and HBV groups based on the GSE10143 dataset (Normal group, *n* = 307; NHBV group, *n* = 61; HBV group, *n* = 19); **(D)** Analysis of the correlation between CASP3 and overall survival of HCC patients by plotting survival curves to analyze the correlation between CASP3 and overall survival of NHBV-related HCC patients (n = 61); **(E)** ROC analysis of the predictive value of CASP3 expression in the overall survival of NHBV-related HCC patients at 1, 3 and 5 years (*n* = 61).

To further investigate the key targets of G. lucidum triterpenes affecting the occurrence and development of NHBV-related HCC. We downloaded the TCGA-LIHC dataset through the TCGA database. We performed differential analysis on CASP3, PTEN, NFKBIA, BCL2, CDKN1A, and TNF expression. The results showed that compared with the Normal group, CASP3, PTEN, and CDKN1A were significantly upregulated in the Tunor group. In contrast, the expression of NFKBIA, BCL2, and TNF was not significantly expressed (Figure 6A). Survival analysis showed that only CASP3 was strongly associated with survival in HCC patients, and the overall survival of patients in the group with high expression of CASP3 was worse (Figure 6B, Supplementary Figure S5A). In addition, our search of the HPA database revealed that CASP3 protein expression was significantly upregulated in cancer samples from HCC patients compared to normal liver tissue (Supplementary Figure S5B).

The previous results of functional enrichment of the gut flora showed that the gut flora of NHBV patients is closely related to apoptosis and p53 pathways. Therefore, we explored the prognostic value of the apoptosis and p53 pathway factors CASP3 in NHBV-related HCC patients. The GSE10143 dataset was retrieved from the GEO database, which contained 307 normal liver tissue samples, 61 liver tissue samples from NHBV-related HCC patients, and 19 liver tissue samples from HBV-related HCC patients. We found that CASP3 expression was significantly upregulated in the NHBV group compared to the Normal group, while there was no significant difference in CASP3 expression in the HBV group (Figures 6C, D). The results of the ROC analysis showed that CASP3 was a good predictor of survival at 1, 3, and 5 years in patients with NHBV-related HCC (Figure 6E).

The above results suggest that CASP3 has a good prognostic value in patients with NHBV-related HCC and that the Apoptosis and p53 pathway factor CASP3 is a key gene mediating the imbalance of intestinal flora for the development and progression of NHBV-related HCC.

## 4 Discussion

In this study, based on the analysis of 16S rRNA sequencing data, we found that the abundance of intestinal flora was significantly higher in both the HBV-related HCC group (HBV group) and the NHBV-related HCC group (NHBV group) than in the HC group (HC group), and there were significant differences in species composition between groups at the phylum and genus levels, with the NHBV-related HCC was more significantly different than HBV-related HCC. Based on genus abundance data, KEGG enrichment analysis of the gut flora revealed that the NHBV group gut flora was mainly enriched in functional pathways such as apoptosis and p53 signalling.

The intestinal tract is the immune organ of the human body, and the intestinal flora is known as the “second human genome”. The intestinal flora regulates the immune response of the host. It is involved in important physiological functions of various systems in the human body, and dysbiosis of the intestinal flora affects the mechanisms of disease development (Chen T et al., 2021). Numerous studies have shown that changes in the intestinal flora play an important role in developing HCC (Lapidot et al., 2020; Zhang et al., 2021). The increasing construction of the global human gut microbial database provides an important basis for exploring the mechanism of action of the intestinal flora.

As the 16S rRNA gene is the most commonly used molecular marker for systematic taxonomic studies of prokaryotic microorganisms, allowing precise quantification of all species in the gut microbiome, 16S rRNA sequencing has become one of the most commonly used high-throughput sequencing-dependent histological technologies (Clarridge, 2004). Chung MW et al. used CLC Genomics Workbench analysis of macrogenomics data from 16S rRNA sequencing, revealed that gut microbiome composition could predict response to nivolumab in patients with advanced HCC (Chung et al., 2021).

It is worth mentioning that Proteobacteria are increased at the portal level in patients with chronic liver disease and HCC compared to healthy individuals (Chen T et al., 2021). In addition, Proteobacteria were increased, and Ruminococcus were decreased in the intestinal flora of HCC patients in the presence of vascular invasion compared to the absence of vascular invasion group (Zhang N et al., 2022). All these results are consistent with the results of this study. It can be seen that investigating the changes in intestinal flora in patients with NHBV-related HCC is valuable to investigate the pathogenesis and improving the prognosis.

Intestinal flora affects tumor progression by influencing the regulation of metabolic pathways and participating in the modulation of key pathways. For example, *Clostridium* butyricum (a butyrate-producing probiotic) inhibits the development of intestinal tumors by regulating Wnt signalling and intestinal flora (Chen et al., 2020). The role of traditional Chinese medicine or natural compounds in intestinal flora regulation and anti-tumor has been gradually demonstrated. The randomized controlled trial results showed that detoxification granules had a modulating effect on the composition of intestinal flora in patients with advanced HCC, which may be closely related to its effect on improving prognosis (Yifu et al., 2022).

G. lucidum is a valuable traditional Chinese herb with excellent anticancer, hepatoprotective, and immunomodulatory properties, and its use as an alternative medicine is becoming increasingly popular among cancer patients (Jin et al., 2016). Reishi has been shown to reduce AOM/DSS-induced colitis and tumourigenesis by inhibiting the TLR4/MyD88/NF-κB pathway, improving intestinal flora dysbiosis, and increasing the production of short-chain fatty acids (Guo et al., 2021). Thus, we speculate: can G. lucidum triterpenes, as the main active component of G. lucidum to exert anti-tumour effects, influence HCC occurrence and development by altering intestinal flora?

In this study, the network pharmacology analysis revealed that 465 G. lucidum triterpenes-related targets were intersected with 4186 HCC-related targets to obtain 176 intersecting targets, among which Apoptosis and p53 pathway factors were located at the core of the protein interactions network of the intersecting targets. This implies that G. lucidum triterpenes may improve the imbalance of intestinal flora through apoptosis and p53 pathways, thereby affecting the occurrence and development of HCC. To date, 316 G. lucidum triterpenes have been identified, and the key components through which G. lucidum triterpenes act need to be better understood (Cor et al., 2018). Therefore, we constructed a “G. lucidum triterpenes component-target-pathway” network based on the correspondence between 13 Apoptosis and p53 pathway-related targets and 10 G. lucidum triterpenes components. We then prepared small molecule ligand files and protein receptor files to identify the active pockets of the receptor proteins and used Vina software to perform molecular docking. We found that Ganosporelactone B, the active component of G. lucidum triterpenes, had the lowest Binding Affinity of −9.3 kal/mol, suggesting that Ganosporelactone B may be the key component of G. lucidum triterpenes affecting the balance of intestinal flora in HCC patients and CASP3 is the key target.

The protein encoded by the CASP3 (caspase 3) gene is a cysteine-aspartate protease that plays a central role in the execution phase of apoptosis (Nagata, 2018) (Supplementary Figure S2). Yu N et al. found that G. lucidum triterpenes reduced neuronal apoptosis, with significant changes in the expression levels of the apoptosis-related proteins Bcl2, Bax, caspase 3/cleaved caspase 3 (Yu et al., 2020). Bax, caspase 3/cleaved caspase 3 expression levels were significantly altered (Yu et al., 2020). In addition, CASP3 is also an important regulator of the p53 pathway (Supplementary Figure S3).

Dihydroartemisinin has been shown to inhibit gastric carcinogenesis and invasion by regulating STAT1/KDR/MMP9 and p53/BCL2L1/CASP3/7 pathways (Liang et al., 2021). Although there is more evidence in the literature that CASP3 plays an important role in tumor cell apoptosis, it is unknown whether CASP3 is involved in regulating intestinal flora. It has been shown that the vanilloid derivative VND3207 protects the intestine from radiation damage by regulating the p53/NOXA pathway and restoring intestinal flora homeostasis (Li et al., 2019). These results suggest that CAPS3 may affect intestinal flora balance in HCC patients by activating apoptosis and p53 pathways.

To further investigate the prognostic value of CASP3 in HCC patients, we performed bioinformatics analysis based on the TCGA-LIHC dataset in the TCGA database and the GSE10143 dataset in the GEO database. The results showed that the mRNA and protein expression of CASP3 was significantly upregulated in cancer tissue samples from HCC patients compared to normal liver tissue samples. CASP3 predicted the survival rate of NHBV-related HCC patients at 1, 3, and 5 years better. The above results will be useful for the precise treatment of NHBV-related HCC patients in the future. It will be valuable for guiding the clinical use of G. lucidum triterpenes.

## 5 Conclusion

**FIGURE 7:**
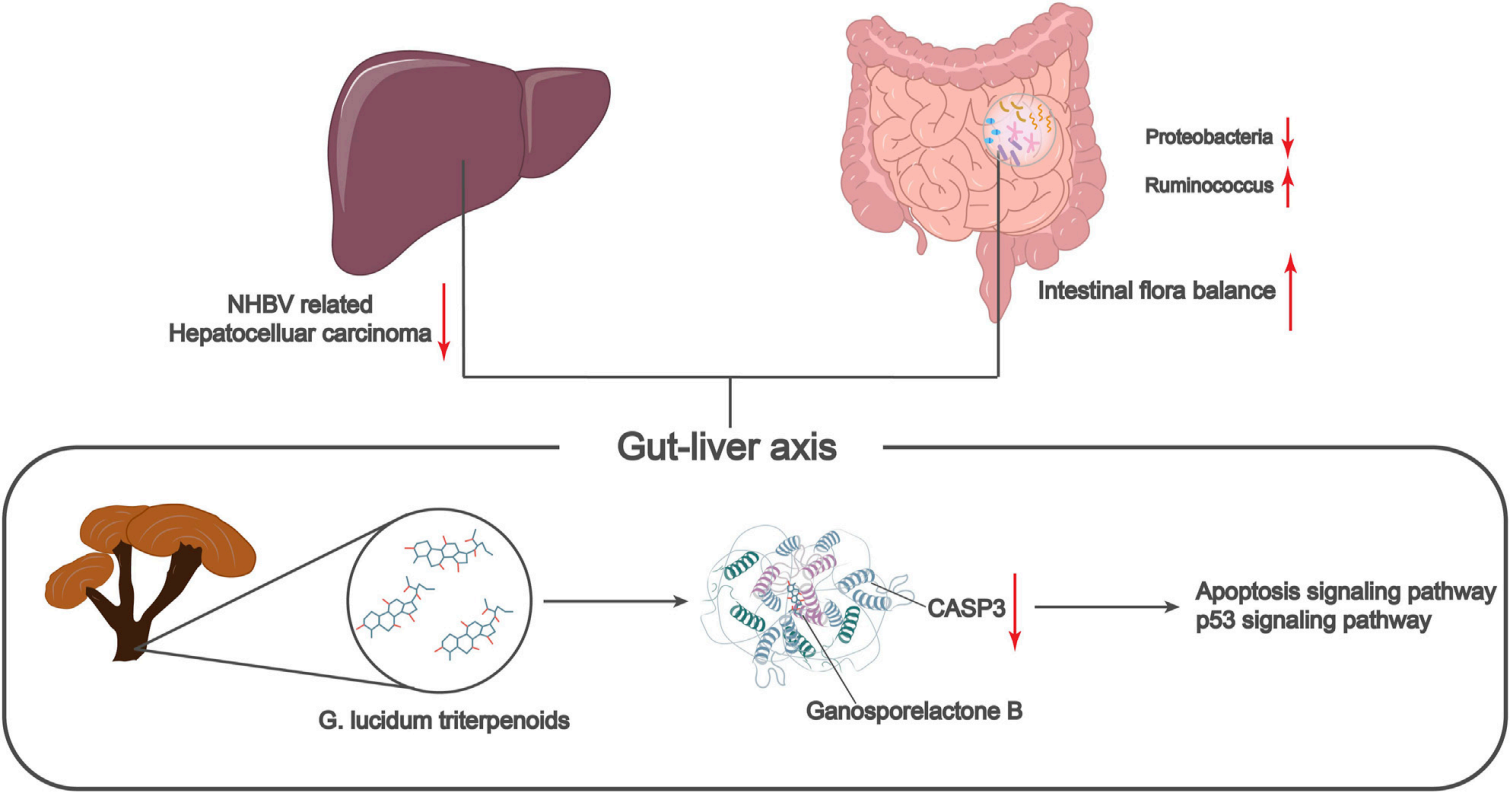
Molecular mechanism of G. lucidum triterpenes to improve intestinal flora imbalance in the treatment of non-HBV-related hepatocellular carcinoma.

In summary, we can tentatively conclude that Ganosporelactone B, the active ingredient of G. lucidum triterpenes, can improve the imbalance of intestinal flora by targeting the activation of apoptosis and p53 pathways through the inhibition of CASP3 and ultimately prevent the occurrence and development of NHBV-related HCC (Figure 7). The present study provides a new theoretical basis and molecular target to reveal the underlying mechanism of G. lucidum triterpenes in treating HCC through the regulation of intestinal flora. However, due to the complexity of the key pathways and the process by which the intestinal flora acts, the current work is limited to public data and needs more experimental validation. In our next work, we will further validate the key components, targets, and pathways of G. lucidum triterpenes affecting HCC through animal and cellular experiments. We will also provide more references for the clinical application of G. lucidum triterpenes.

## Data Availability

The original contributions presented in the study are included in the article/Supplementary Material, further inquiries can be directed to the corresponding authors.

## Supplementary material

SUPPLEMENTARY FIGURE S1 Comparison of the abundance of intestinal flora between normal control and HCC patients Note: **(A)** Volcano plot comparing the difference in abundance between the HC and NHBV groups; **(B)** Heat map comparing the difference in abundance between the HC and NHBV groups; **(C)** Manhattan plot comparing the difference in abundance between the HC and NHBV groups; **(D)** Volcano plot comparing the difference in abundance between the HC and HBV groups; **(E)** Heat map comparing the difference in abundance between the HC and HBV groups; **(F)** Manhattan plot comparing the difference in abundance between the HC and HBV groups. **(F)** Manhattan plot of the difference in abundance between the HC and HBV groups; in Figures A and D, red dots indicate enriched, green dots indicate depleted, grey dots indicate non-significant, each dot represents an individual OTU, and the values in the plots indicate the number of OTUs that are differentially enriched; in Figures C and F, the different colors represent the different intestinal flora at the gate level; HC group, n = 33; NHBV group, n = 22; HBV group, n = 22 22; HBV group, n = 35.

SUPPLEMENTARY FIGURE S2 Diagram of the regulatory mechanism of apoptosis pathway (hsa04210) Note: The pathway factors marked with red pentagrams are the intersecting targets of G. lucidum triterpenes and HCC.

SUPPLEMENTARY FIGURE S3 p53 pathway regulatory mechanism (hsa04115) Note: The pathway factors marked with red pentagrams are the intersecting targets of G. lucidum triterpenes and HCC.

SUPPLEMENTARY FIGURE S4 Molecular docking of each small molecule ligand to the protein receptor Note: The blue part of the diagram is the protein receptor, and the green part is the small molecule ligand.

SUPPLEMENTARY FIGURE S5 Correlation of CASP3 protein expression and other 5 genes with survival in HCC patients Note: **(A)** Correlation between PTEN, NFKBIA, BCL2, CDKN1A, TNF, and overall survival of HCC patients by plotting survival curves (n = 371); **(B)** Protein expression of CASP3 in normal liver tissue samples and cancer tissue samples from HCC patients.
