# Supplementary figures and images for: G. lucidum triterpenes restores intestinal flora balance in non-hepatitis B virus-related hepatocellular carcinoma: evidence of 16S rRNA sequencing and network pharmacology analysis
Source: Front Pharmacol. 2023 Sep 18;14:1197418. doi: 10.3389/fphar.2023.1197418 (PMC10544910; doi:10.3389/fphar.2023.1197418)

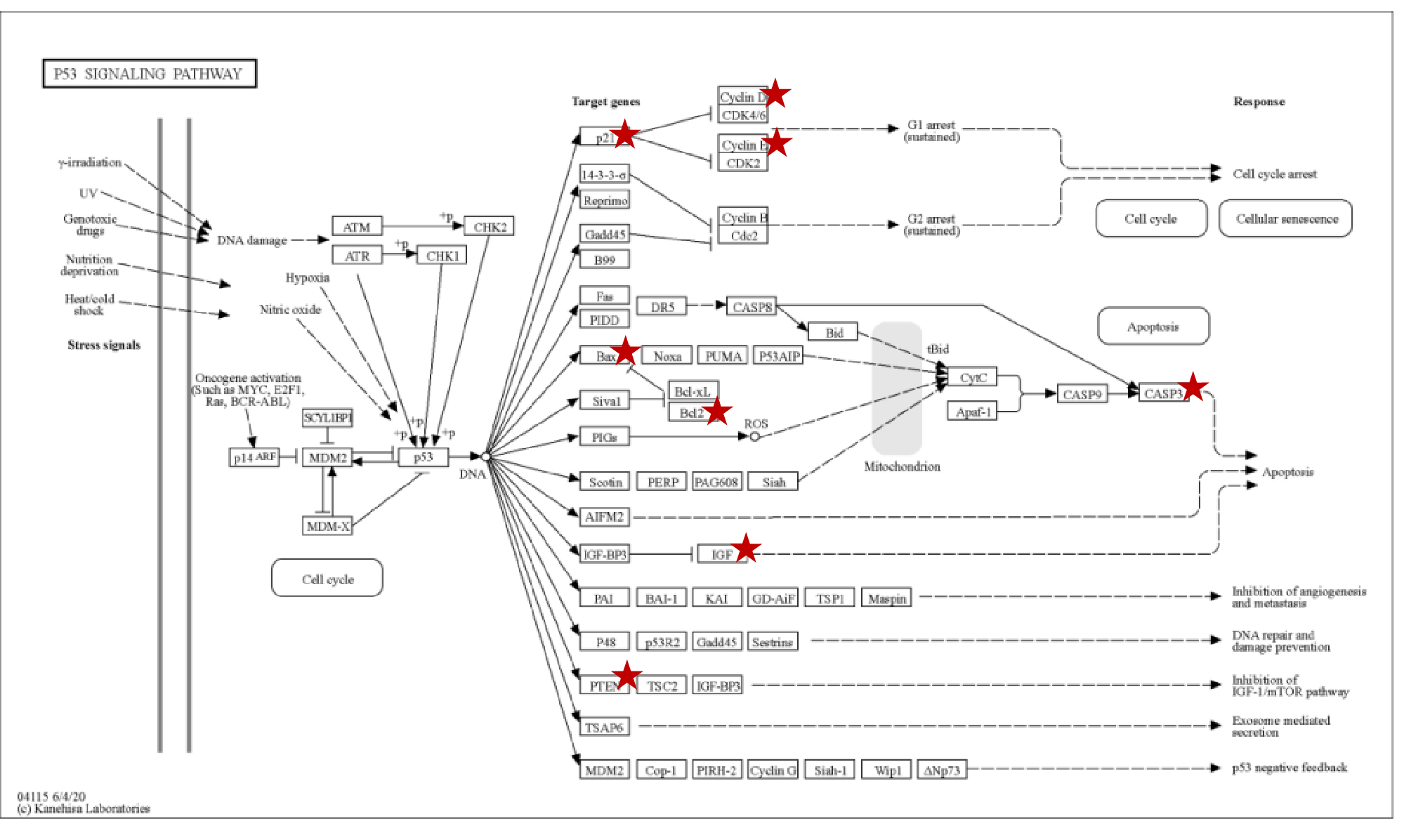

Supplement: Supplementary file 1 [file Image3.JPEG]

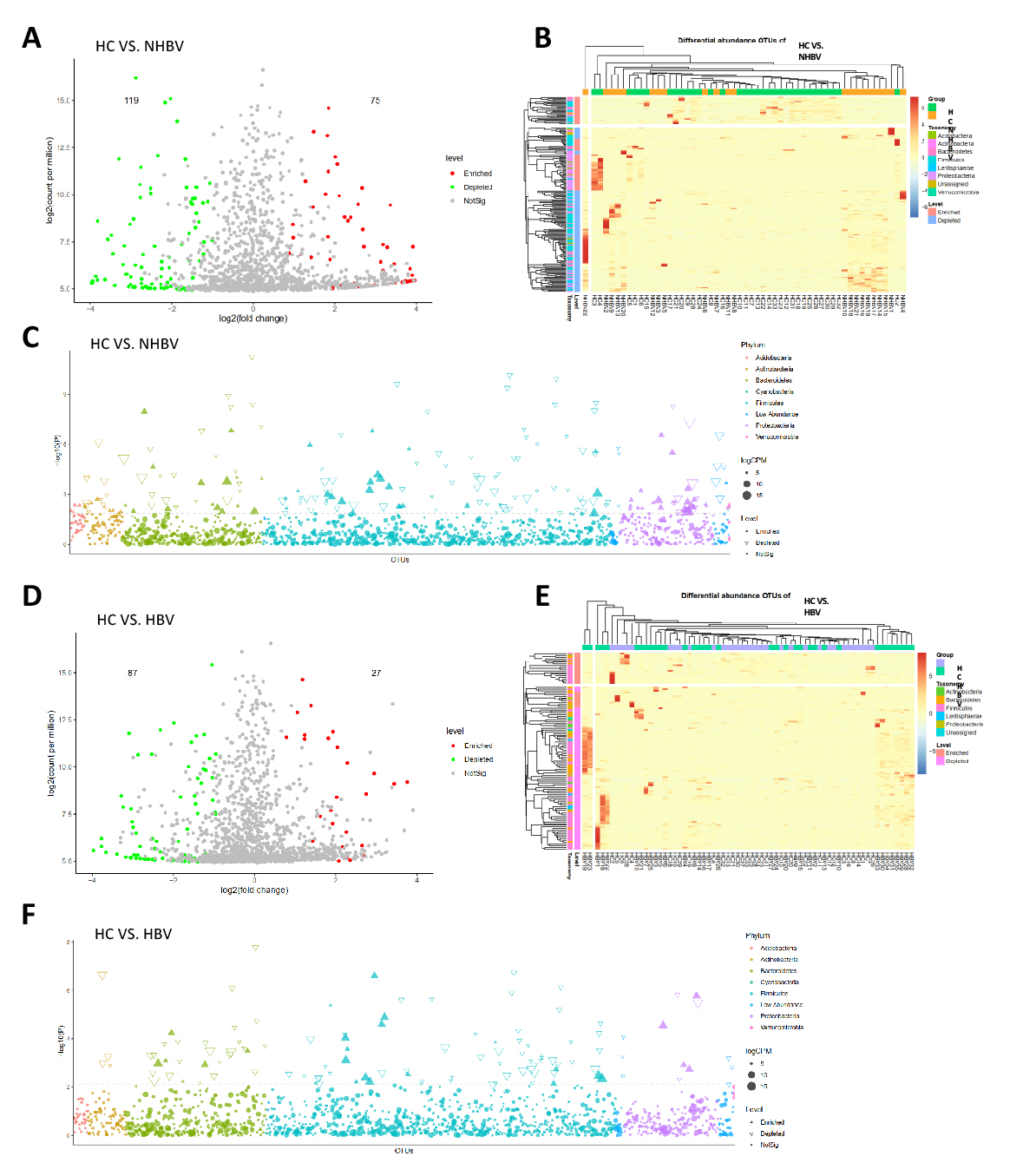

Supplement: Supplementary file 3 [file Image1.JPEG]

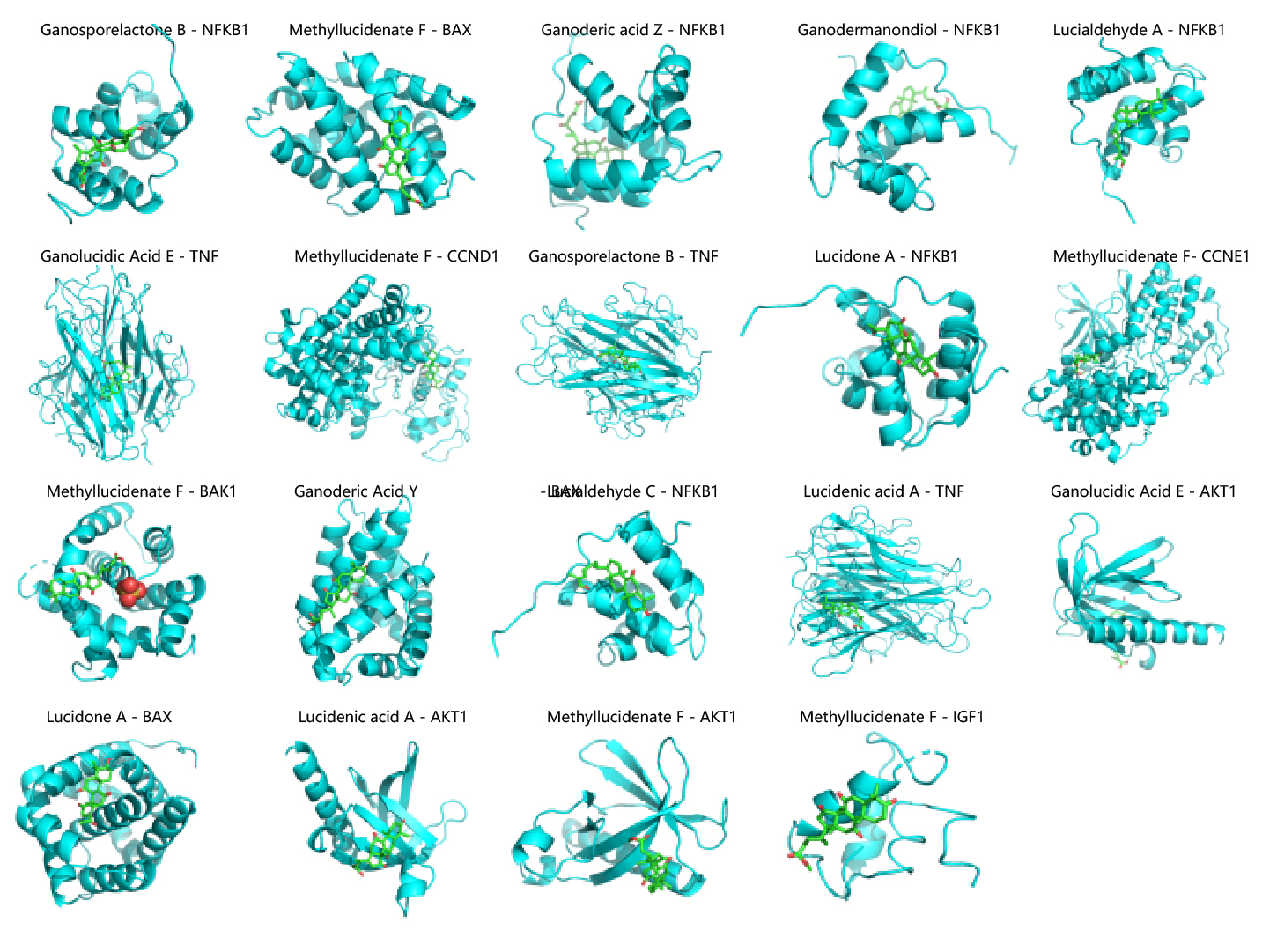

Supplement: Supplementary file 4 [file Image4.JPEG]

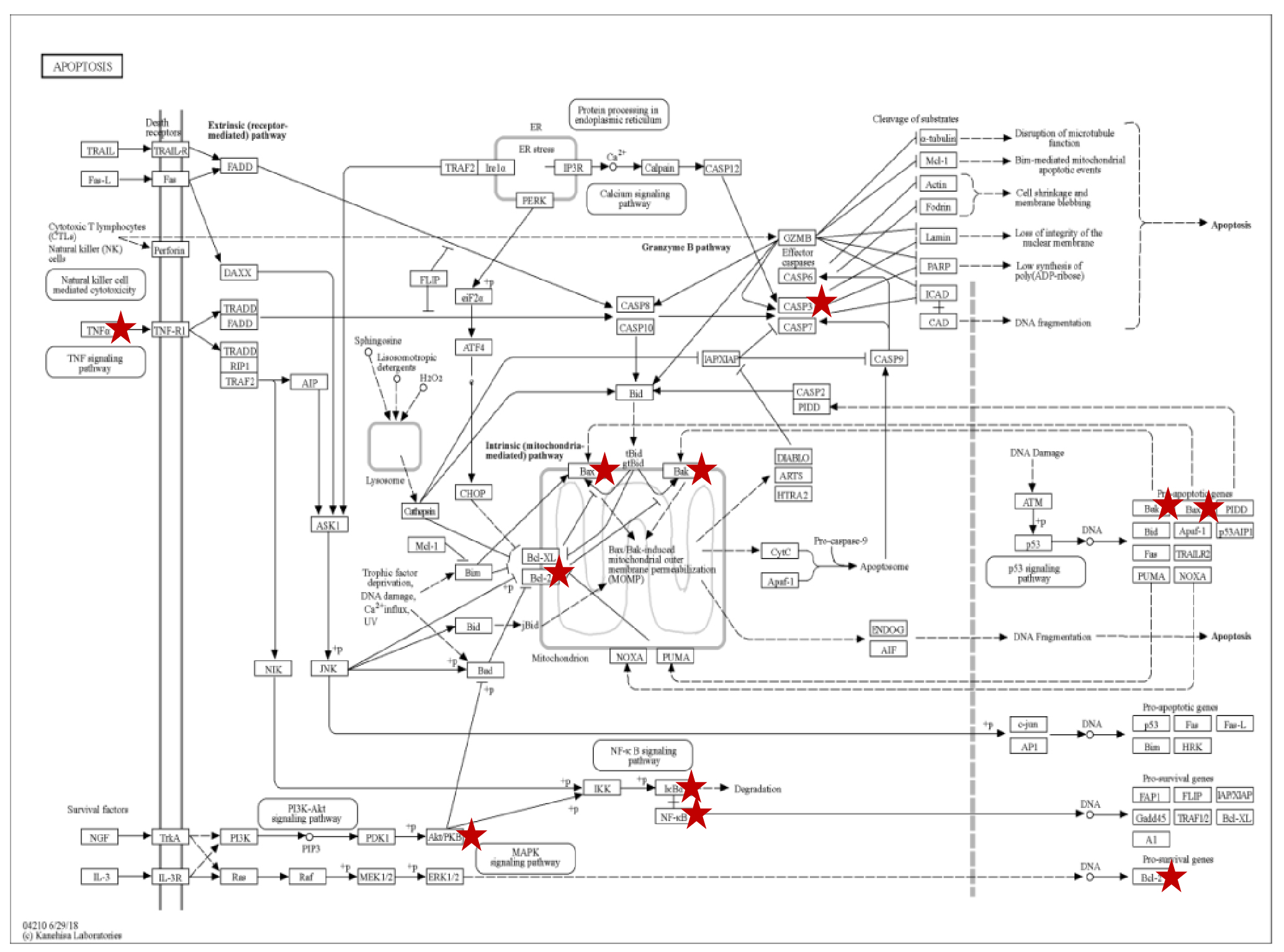

Supplement: Supplementary file 5 [file Image2.JPEG]

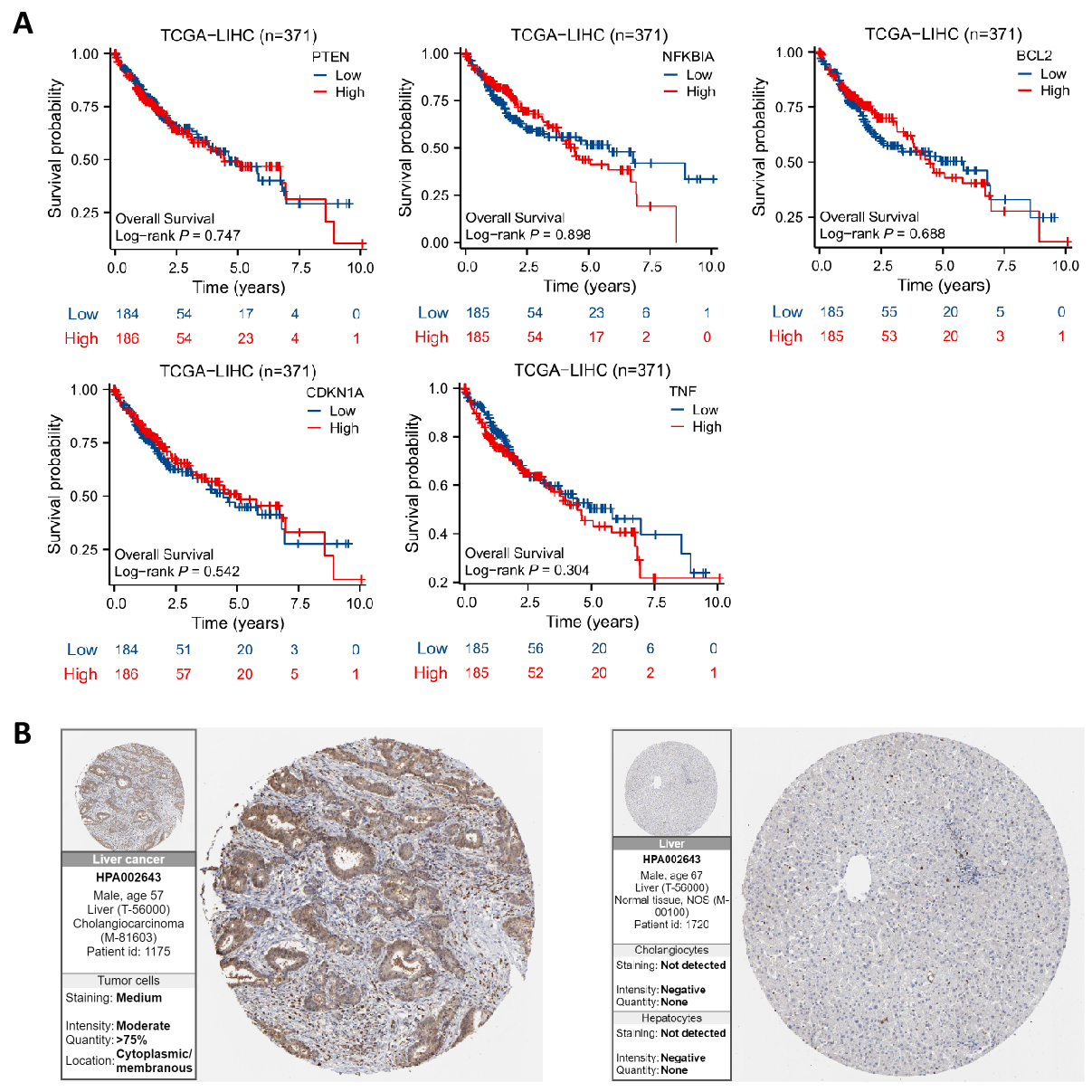

Supplement: Supplementary file 6 [file Image5.JPEG]
