# Supplementary material for: G. lucidum triterpenes restores intestinal flora balance in non-hepatitis B virus-related hepatocellular carcinoma: evidence of 16S rRNA sequencing and network pharmacology analysis
Source: Front Pharmacol. 2023 Sep 18;14:1197418. doi: 10.3389/fphar.2023.1197418 (PMC10544910; doi:10.3389/fphar.2023.1197418)
Supplement: Supplementary file 2 [file Table1.DOCX]

**Table S1 Constituents of Ganoderma lucidum triterpenes**

| **No.** | **MOL ID** | **PubChem CID** | **Synonyms** | **Molecular Formula** | **IUPAC Name** | **Isomeric SMILES** | **MW (g/mol)** | **OB** | **ALOGP** | **HDON** | **HACC** |
| --- | --- | --- | --- | --- | --- | --- | --- | --- | --- | --- | --- |
| 1 | MOL011241 | 73294 | Ganodermanondiol | C30H48O3 | (5R,10S,13R,14R,17R)-17-[(2R,5S)-5,6-dihydroxy-6-methylheptan-2-yl]-4,4,10,13,14-pentamethyl-1,2,5,6,12,15,16,17-octahydrocyclopenta[a]phenanthren-3-one | C[C@H](CC[C@@H](C(C)(C)O)O)[C@H]1CC[C@@]2([C@@]1(CC=C3C2=CC[C@@H]4[C@@]3(CCC(=O)C4(C)C)C)C)C | 456.78 | 37.64 | 5.57 | 2 | 3 |
| 2 | MOL011268 | 10366713 | Lucialdehyde C | C30H46O3 | (E,6R)-6-[(3S,5R,10S,13R,14R,17R)-3-hydroxy-4,4,10,13,14-pentamethyl-7-oxo-1,2,3,5,6,11,12,15,16,17-decahydrocyclopenta[a]phenanthren-17-yl]-2-methylhept-2-enal | C[C@H](CC/C=C(\C)/C=O)[C@H]1CC[C@@]2([C@@]1(CCC3=C2C(=O)C[C@@H]4[C@@]3(CC[C@@H](C4(C)C)O)C)C)C | 454.76 | 42.26 | 6.14 | 1 | 3 |
| 3 | MOL011226 | 10601916 | Ganoderic acid Z | C30H42O7 | (E,6R)-6-[(3S,5R,10S,13R,14R,17R)-3-hydroxy-4,4,10,13,14-pentamethyl-2,3,5,6,7,11,12,15,16,17-decahydro-1H-cyclopenta[a]phenanthren-17-yl]-2-methylhept-2-enoic acid | C[C@H](CC/C=C(\C)/C(=O)O)[C@H]1CC[C@@]2([C@@]1(CCC3=C2CC[C@@H]4[C@@]3(CC[C@@H](C4(C)C)O)C)C)C | 456.78 | 37.67 | 7.24 | 2 | 3 |
| 4 | MOL011266 | 11048424 | Lucialdehyde A | C30H46O2 | (E,6R)-6-[(3S,5R,10S,13R,14R,17R)-3-hydroxy-4,4,10,13,14-pentamethyl-2,3,5,6,12,15,16,17-octahydro-1H-cyclopenta[a]phenanthren-17-yl]-2-methylhept-2-enal | C[C@H](CC/C=C(\C)/C=O)[C@H]1CC[C@@]2([C@@]1(CC=C3C2=CCC4[C@@]3(CC[C@@H](C4(C)C)O)C)C)C | 438.76 | 44.78 | 6.84 | 1 | 2 |
| 5 | MOL011270 | 14109375 | Lucidenic acid A | C27H38O6 | (4R)-4-[(5R,7S,10S,13R,14R,17R)-7-hydroxy-4,4,10,13,14-pentamethyl-3,11,15-trioxo-1,2,5,6,7,12,16,17-octahydrocyclopenta[a]phenanthren-17-yl]pentanoic acid | C[C@H](CCC(=O)O)[C@H]1CC(=O)[C@@]2([C@@]1(CC(=O)C3=C2[C@H](C[C@@H]4[C@@]3(CCC(=O)C4(C)C)C)O)C)C | 458.65 | 30.34 | 2.48 | 2 | 6 |
| 6 | MOL011256 | 15602283 | Ganolucidic Acid E | C30H44O5 | (E,6R)-6-[(5R,10S,13R,14R,15S,17R)-15-hydroxy-4,4,10,13,14-pentamethyl-3,11-dioxo-2,5,6,7,12,15,16,17-octahydro-1H-cyclopenta[a]phenanthren-17-yl]-2-methylhept-2-enoic acid | C[C@H](CC/C=C(\C)/C(=O)O)[C@H]1C[C@@H]([C@@]2([C@@]1(CC(=O)C3=C2CC[C@@H]4[C@@]3(CCC(=O)C4(C)C)C)C)C)O | 484.74 | 32.85 | 4.96 | 2 | 5 |
| 7 | MOL011129 | 21633085 | Methyl lucidenate F | C28H38O6 | methyl (4R)-4-[(5R,10S,13R,14R,17R)-4,4,10,13,14-pentamethyl-3,7,11,15-tetraoxo-2,5,6,12,16,17-hexahydro-1H-cyclopenta[a]phenanthren-17-yl]pentanoate | C[C@H](CCC(=O)OC)[C@H]1CC(=O)[C@@]2([C@@]1(CC(=O)C3=C2C(=O)C[C@@H]4[C@@]3(CCC(=O)C4(C)C)C)C)C | 470.66 | 32.67 | 2.69 | 0 | 6 |
| 8 | MOL011225 | 57397445 | Ganoderic Acid Y | C30H46O3 | (E,6R)-6-[(3S,5R,10S,13R,14R,17R)-3-hydroxy-4,4,10,13,14-pentamethyl-2,3,5,6,12,15,16,17-octahydro-1H-cyclopenta[a]phenanthren-17-yl]-2-methylhept-2-enoic acid | C[C@H](CC/C=C(\C)/C(=O)O)[C@H]1CC[C@@]2([C@@]1(CC=C3C2=CC[C@@H]4[C@@]3(CC[C@@H](C4(C)C)O)C)C)C | 454.76 | 38.64 | 6.79 | 2 | 3 |
| 9 | MOL011287 | 71453988 | Lucidone A | C24H34O5 | (3S,5R,7S,10S,13R,14R,17S)-17-acetyl-3,7-dihydroxy-4,4,10,13,14-pentamethyl-2,3,5,6,7,12,16,17-octahydro-1H-cyclopenta[a]phenanthrene-11,15-dione | CC(=O)[C@H]1CC(=O)[C@@]2([C@@]1(CC(=O)C3=C2[C@H](C[C@@H]4[C@@]3(CC[C@@H](C4(C)C)O)C)O)C)C | 402.58 | 37.22 | 1.09 | 2 | 5 |
| 10 | MOL011258 | 78384958 | Ganosporelactone B | C30H42O7 | 10',16',20'-trihydroxy-2',3,7',9',13',17',17'-heptamethylspiro[oxolane-5,5'-pentacyclo[10.8.0.02,9.04,8.013,18]icos-1(12)-ene]-2,3',11'-trione | C[C@@H]1C[C@@]2(C[C@H](C(=O)OCC3([C@H](CC[C@]4(C3C[C@H](C5=C4C(=O)[C@H]([C@@]6(C1[C@@H]2C(=O)[C@@]56C)C)O)O)C)O)C)C)O | 530.72 | 31.21 | 1.22 | 4 | 8 |

Notes: MW, Molecular Weight; OB, Oral bioavailability; ALOGP, Lipid/ waterPartition Coeffici; HDON, Hydrogen-bonding donor; HACC, Hydrogen-bonding acceptor

**Table S2 Molecular docking results of Ganoderma lucidum triterpene small molecule ligand and protein receptor**

| Molecular ligand | Protein receptor | Binding Affinity (kcal/mol ) |
| --- | --- | --- |
| Ganosporelactone B | CASP3 | -9.3 |
| Methyllucidenate F | PTEN | -9 |
| Methyllucidenate F | NFKBIA | -8.9 |
| Ganoderic Acid Y | BCL2 | -8.6 |
| Methyllucidenate F | CDKN1A | -8.1 |
| Ganoderic acid Z | TNF | -8 |
| Ganosporelactone B | NFKB1 | -7.8 |
| Methyllucidenate F | BAX | -7.7 |
| Ganoderic acid Z | NFKB1 | -7.7 |
| Ganodermanondiol | NFKB1 | -7.6 |
| Lucialdehyde A | NFKB1 | -7.6 |
| Ganolucidic Acid E | TNF | -7.6 |
| Methyllucidenate F | CCND1 | -7.5 |
| Ganosporelactone B | TNF | -7.3 |
| Lucidone A | NFKB1 | -7.2 |
| Methyllucidenate F | CCNE1 | -7.1 |
| Methyllucidenate F | BAK1 | -6.9 |
| Ganoderic Acid Y | BAX | -6.8 |
| Lucialdehyde C | NFKB1 | -6.8 |
| Lucidenic acid A | TNF | -6.8 |
| Ganolucidic Acid E | AKT1 | -6.7 |
| Lucidone A | BAX | -6.7 |
| Lucidenic acid A | AKT1 | -6.3 |
| Methyllucidenate F | AKT1 | -6.3 |
| Methyllucidenate F | IGF1 | -5.8 |
